# Prevalence and risk factors of hepatitis B infection in rural communities: a cross-sectional analysis

**DOI:** 10.3389/fpubh.2026.1889741

**Published:** 2026-10-13

**Authors:** Prospera Asakitogum, Francis Anaba Agana, Comfort Dede, Victoria Loerzel, David Ayangba Asakitogum

**Affiliations:** 1 Ataribuno Cervix Foundation, Bolgatanga, Ghana; 2 Presbyterian Primary Health Care, Bolgatanga, Ghana; 3 School of Nursing and Midwifery, University of Technology and Applied Sciences, Navrongo, Ghana; 4 Midwifery Training College, Bolgatanga, Ghana; 5 College of Nursing, Academic Health Sciences Center, University of Central Florida, Orlando, FL, United States

**Keywords:** adults, communities, Ghana, HBV, infection, prevalence, rural

## Abstract

**Objective:**

Hepatitis B virus (HBV) infection is a highly contagious preventable liver disease with high mortality rates across the world. HBV is endemic in Ghana, yet screening for early detection and treatment of the condition is a challenge. This study assessed the prevalence and associated risk factors of HBV infection in rural communities in the Upper East Region of Ghana.

**Subjects and methods:**

This is a cross-sectional analysis of secondary data from 218 participants. The participants were classified into 2 groups—HBV negative and positive groups. Fisher Exact and Chi Square analysis were used to evaluate association between hepatitis B infection status and demographic and clinical characteristics. Binary logistic regression was used to evaluate for the risk factors associated with the HBV positive group.

**Results:**

The mean (SD) age of the participants was 39.04(13.65) years, with majority 18–30 years old (30.28%), and 55.50% were farmers. The prevalence of HBV infection was 9.63%. Farmers were less likely associated with risk of HBV infections (OR = 0.17; 95% CI: 0.052–0.529; *p* = 0.002) than traders. After adjusting for other confounders, participants who had handwork were associated with lower risk of HBV infections (OR = 0.14; 95% CI: 0.028–0.738; *p* = 0.020) than traders. No family history of hepatitis B was associated with lower risk of HBV infections (OR = 0.09; 95% CI: 0.021–0.416; *p* = 0.002) than participants with family history of hepatitis B.

**Conclusion:**

We found high prevalence of HBV infection among rural adults in this study. Associated risk factors identified for transmission of HBV infection in this population included occupation and family history of hepatitis B infection. There is urgent need for the development of context-specific prevention strategies that address both social and household transmission dynamics of HBV in rural communities in Ghana.

## 1 Introduction

Hepatitis B virus (HBV) infection remains a major global public health concern, which contributes substantially to liver-related morbidity and mortality worldwide (1). An estimated 316 million people are chronically infected with HBV (2). The highest burden of HBV infection is borne by low- and middle- income countries, particularly in sub-Saharan Africa (1). The virus is highly infectious and can be transmitted through contact with infected body fluids and/ or blood/ transfusion (3–5), sexual contact (6), perinatal transmission (7), and the use of contaminated sharp instruments (5, 8). However, there are effective HBV vaccines (e.g., Heplisav-B) that has highest seroprotection rates (99.4%) with two doses (9) for the prevention of HBV infection. A major challenge in the control of HBV infection is that most infected individuals remain asymptomatic for long periods, which facilitates silent transmission within communities (10).

Africa continues to experience a disproportionately high prevalence of the HBV infection compared with other regions of the world (1). This burden is compounded by limited access to screening, delayed diagnosis, inadequate vaccination coverage among adults, and persistent socio-cultural practices that increase exposure risk (11, 12). In many African settings, HBV testing is not routinely integrated into primary healthcare services, especially in rural areas (1), allowing infections to go undetected until advanced liver disease develops. As a result, HBV remains a major contributor to chronic hepatitis, liver cirrhosis, and hepatocellular carcinoma (13).

In Ghana, HBV infection is considered hyperendemic, with nationwide seroprevalence of 8.48% (11) which consistently exceed the World Health Organization’s threshold of >2% for high endemicity (1). Evidence indicates marked regional variations, with the Upper East Region (UER) ranked third (12.04%) among the areas with the highest prevalence of HBV infection (14). Despite this, most existing studies have focused on urban populations (15, 16), healthcare workers or occupational groups (3, 17), and pregnant women (7, 18), leaving rural communities relatively understudied. Rural populations are particularly vulnerable due to limited health infrastructure, lower health literacy, higher levels of poverty, and increased engagement in traditional practices such as scarification, traditional circumcision, and home-based healthcare procedures (12, 19, 20), which may facilitate HBV transmission.

Common socio-demographic risk factors include sex, marital status, education, occupation, and household contact (11, 21, 22). The behavioral-related risk factors identified were intravenous drug use (23), history of blood transfusion, sexually transmitted infections (STIs), and multiple sexual partners (5, 6, 22). However, the relative contribution of these risk factors within rural communities of the UER remains poorly characterized. Understanding these localized risk patterns is essential for designing effective and context-specific prevention and control strategies. Therefore, the aim of this study was to assess the prevalence and associated risk factors of HBV infection in rural communities in the UER of Ghana.

## 2 Materials and methods

### 2.1 Study design and setting

This study is a cross-sectional analysis of secondary data from the Bembisi Health Screening (BHS) dataset collected annually between March and June since 2024. The data is hosted by the Kassena Nankana East Health Center (KNE-HC) in Kandiga in the Kassena Nankana West District (KNWD). The primary purpose of the BHS is to identify major health problems in the communities for health education and to encourage regular health screening practices. The 2025 BHS deidentified data was used for the analysis in this study.

The KNWD is a rural district in the UER of Ghana. The district has an estimated population of approximately 90,735 people, with 79.6% residing in the rural areas (24). The district has 6 Health Centers and 28 functional Community based Health and Planning Services (CHPS) centers with no district hospital. Although English is the official language, the Kassena primarily speak Kasem, while the Nankana communicate in Gurune. The data collection was conducted in English and Gurune.

### 2.2 Sampling technique

The BHS employed a community-based voluntary participation approach. Community members from the two traditional areas served by the KNE-HC were mobilized through community announcements and invited to participate in the annual screening exercise. For this secondary analysis, a census sampling approach was used whereby all available records from the 2025 BHS database were screened for eligibility. Participants were included if they were aged ≥18 years or older, resided in the study communities, participated in the 2025 BHS, and had complete data on variables of interest. Individuals younger than 18 years (might have received childhood HB immunization), visitors to the communities, patients already registered at the KNE-HC, and records with incomplete data were excluded. Of the 413 individuals screened, 218 met the eligibility criteria and were included in the final analysis.

Because this study was based on secondary analysis of an existing dataset, no formal sample size calculation was conducted prior to the study. Instead, all eligible records from the 2025 BHS database were considered for inclusion. Including all eligible participants enhanced the representativeness of the available dataset and maximized the statistical power of the study.

### 2.3 Instrument

This study utilized secondary data obtained from the 2025 BHS database. During the original BHS screening exercise, data were collected using the Bembisi Community Health Screening Questionnaire (BCHSQ), a structured questionnaire administered to eligible participants by trained screening personnel. The BCHSQ captured information on sociodemographic characteristics, clinical history, and risk factors for hypertension, diabetes, HBV infection, and anemia (Supplementary Sheet 1). For the present study, deidentified questionnaire responses and HBV screening results from the 2025 BHS database were extracted and analyzed. No additional data were collected from participants for this secondary analysis.

The participants’ HBV status was measured using the HIGHTOP HBsAg Test Kit (Qingdao HIGHTOP Biotech Co. Ltd., Qingdao, China), a rapid immunoassay test which identifies current or past infection or absent of infection in human serum, plasm or whole blood. The test was conducted by a professional laboratory technician, and results of the test was recorded as either HBV negative or positive case. HBV profile test was done for all positive cases, and they were referred for medical advice and treatment. All the negative cases received first dose HBV vaccine (EUVAX with batch #WVA24503) (LG Chem Ltd., Seoul, South Korea), and they were follow-up to complete the scheduled vaccination.

### 2.4 Variables

HBV status was assessed as the dependent variable. The selected socio-demographic characteristics in this analysis included the participants’ age, sex, marital status, level of education, occupation, and financial status. HBV risk-related independent variables included were family history of liver disease or jaundice, sharing of bath towel and toothbrush with other family members, history of STIs or having multiple sexual partners, history of blood transfusion, and alcohol consumption.

### 2.5 Data analysis

The data was analyzed using STATA/BE version 17.0 (25). Descriptive statistics, including means, standard deviations for continues quantitative variables and frequencies and percentages for categorical variables were generated to describe participants characteristics. The HBV status of the participants was recoded into 2 groups—HBV negative (0) and positive (1) groups. Fisher Exact and Chi Square analysis were used to evaluate for differences in demographic and clinical characteristics between the HBV negative and positive groups. Variables identified as risk factors in the literature review (i.e., age, sex, marital status, family history of HBV, history of STIs, occupation, multiple sexual partners) (5, 6, 11, 21, 22) were included in the multivariable models. Binary logistic regression was used to evaluate for the risk factors associated with the HBV positive group. For all the regression analysis, unadjusted and adjusted values (i.e., odds ratios (OR) and their corresponding 95% confidence intervals) were generated. A *p*-value of ≤0.05 was considered statistically significant.

### 2.6 Ethics approval and consent to participate

The study was conducted in accordance with the Declaration of Helsinki for research involving non-human subjects. Ethical approval for the study was obtained from the University of Central Florida Institutional Review Board with reference number STUDY00008638. Permission was also obtained from the KNE-HC Physician Assistant In-charge and deidentified data was granted.

## 3 Results

### 3.1 Socio-demographic characteristics of the participants

A total of 218 participants were included in this study. The mean (SD) age of the participants was 39.04(13.65) years. Majority of the participants were 18–30 years old (30.28%), female (68.81%), had a non-formal education (36.24%), and married (78.90%). In addition, the majority (59.17%) described their financial status as less than adequate, 55.50% were farmers, 23.39% and 36.70% stated they share bath towel with others and had multiple sexual partners, respectively. The prevalence of HBV infection in this sample was 9.63% (Table 1).

**Table 1:** Participants demographic characteristics.

Variable	Mean	SD
Age	39.04	13.66
	*n*	%
Age in groups (years)		
18–30	66	30.28
31–40	63	28.90
41–50	49	22.48
≥51	40	18.34
Hepatitis B status		
Negative	197	90.37
Positive	21	9.63
Sex		
Male	68	31.19
Female	150	68.81
Marital status		
Single	35	16.06
Married	172	78.90
Separated	11	5.04
Educational level		
No formal education	79	36.24
Grade 1–6 (Primary)	50	22.94
Grade 7–9 (JSS/JHS)	58	26.61
Grade 10–12 (SSS/SHS)	24	11.01
Tertiary	7	3.20
Financial status		
Much less than adequate	45	20.64
Less than adequate	129	59.17
Just enough	42	19.27
More than adequate	2	0.92
Occupation		
Trader	26	11.93
Handwork	39	17.89
Government worker	7	3.21
Farmer	121	55.50
Unemployed	19	8.72
Student	6	2.75
Alcohol consumption		
Yes	78	35.78
No	140	64.22
Share bath towel with others		
Yes	51	23.39
No	167	76.61
Share toothbrush with others		
Yes	5	2.29
No	213	97.71
Been diagnosed of STIs		
Yes	56	25.69
No	162	74.31
Family history of hepatitis B		
Yes	16	7.34
No	202	92.66
History transfused blood		
Yes	23	10.55
No	195	89.45
Have multiple sexual partners		
Yes	80	36.70
No	138	63.30

SD, standard deviation; STIs, sexually transmitted infections.

### 3.2 Differences between hepatitis B status and participants socio-demographic characteristics

In Fisher Exact and Chi Square analysis, there was no statistically significant differences among participants in the two groups (i.e., HBV negative and positive) and socio-demographic variables except for occupation (*p* = 0.021) and family history of hepatitis B (*p* = 0.011) (Table 2).

**Table 2:** Fisher exact and Chi Square analysis of differences associated between hepatitis B infection status and demographic and clinical characteristics.

Variables	Hepatitis B status			Statistics
	Total	Negative	Positive	
	n (%)			
Age in groups (years)				
15–30	66 (30.28)	59 (29.95)	7 (33.33)	X^2^ = 1.2346 *p* = 0.745
31–40	63 (28.90)	56 (28.43)	7 (33.33)	
41–50	49 (22.48)	44 (22.34)	5 (23.81)	
50 and above	40 (18.34)	38 (19.29)	2 (9.52)	
Sex				
Male	68 (31.19)	59 (29.95)	9 (42.86)	*p* = 0.226
Female	150 (68.81)	138 (70.05)	12 (57.14)	
Marital status				
Single	35 (16.06)	32 (16.24)	3 (14.29)	X^2^ = 0.0614 *p* = 0.970
Married	172 (78.90)	155 (78.68)	17 (80.95)	
Separated	11 (5.04)	10 (5.05)	1(4.76)	
Educational level				
No formal education	79 (36.24)	73 (37.06)	6 (28.57)	X^2^ = 3.4219 *p* = 0.490
Grade 1–6 (Primary)	50 (22.94)	44 (22.34)	6 (28.57)	
Grade 7–9 (JSS/JHS)	58 (26.61)	50 (25.38)	8 (38.10)	
Grade 10-12(SSS/SHS)	24 (11.01)	23 (11.68)	1(4.76)	
Tertiary	7 (3.20)	7 (3.55)	0 (0)	
Financial status				
Less than adequate	45 (20.64)	45 (22.84)	0 (0)	X^2^ = 6.8733 *p* = 0.076
Just enough	129 (59.17)	112 (56.85)	17 (80.95)	
More than adequate	42 (19.27)	38 (19.29)	4 (19.05)	
Much more than	2 (0.92)	2 (1.02)	0 (0)	
Occupation				
Trader	26 (11.93)	19 (9.64)	7 (33.33)	X^2^ = 13.217 *p* = 0.021*
Handwork	39 (17.89)	35 (17.77)	4 (19.05)	
Government worker	7 (3.21)	7 (3.55)	0 (0)	
Farmer	121 (55.50)	114 (57.87)	7 (33.33)	
Unemployed	19 (8.72)	16 (8.12)	3 (14.29)	
Student	6 (2.75)	6 (3.05)	0 (0)	
Share bath towel with others				
Yes	51 (23.39)	45 (22.84)	6 (28.57)	*p* = 0.589
No	167 (76.61)	152 (77.16)	15 (71.43)	
Share toothbrush with others				
Yes	5 (2.29)	5 (2.54)	0 (0)	*p* = 1.000
No	213 (97.71)	192 (97.46)	21 (100)	
History of sexually transmitted infections				
Yes	56 (25.69)	50 (25.38)	6 (28.57)	*p* = 0.794
No	162 (74.31)	147 (74.62)	15 (71.43)	
Family history of hepatitis B				
Yes	16 (7.34)	11 (5.58)	5 (23.81)	*p* = 0.011*
No	202 (92.66)	186 (94.42)	16 (76.19)	
History of blood transfusion				
Yes	23 (10.55)	21 (10.66)	2 (9.52)	*p* = 1.000
No	195 (89.45)	176 (89.34)	19 (90.48)	
Multiple sexual partners				
Yes	80 (36.70)	73 (37.06)	7 (33.33)	*p* = 0.815
No	138 (63.30)	124 (62.94)	14 (66.67)	
History of alcohol use				
Yes	78 (35.78)	72 (36.55)	6 (28.57)	*p* = 0.319
No	140 (64.22)	125 (63.45)	15 (71.43)	

FE, fisher exact. *Significant @ ≤ 0.05.

### 3.3 Associated risk factors with hepatitis B infection in rural dwellers

For the unadjusted model, there were statistically significant lower associated risk of hepatitis B infections (OR = 0.17; 95% CI: 0.052–0.529; *p* = 0.002) in participants who were farmers compared to those who were traders. Compared to participants with family history of hepatitis B, there were statistically significant lower associated risk of hepatitis B infections (OR = 0.19; 95% CI: 0.059–0.612; *p* = 0.005) in those without a family history of hepatitis B (Table 3).

**Table 3:** Binary logistic regression of risk factors for Hepatitis B infection in rural dwellers (unadjusted and adjusted).

Variables	Hepatitis B infection							
	Unadjusted				Adjusted			
	Odds	95% CI		*p*-value	Odds	95% CI		*p*-value
Age in groups (years)								
15–30*	1				1			
31–40	1.05	0.437	3.196	0.927	0.41	0.091	1.818	0.239
41–50	0.96	0.285	3.219	0.944	1.42	0.302	6.688	0.658
51 and above	0.44	0.087	2.249	0.326	0.36	0.052	2.469	0.297
Sex								
Male*	1				1			
Female	0.57	0.228	1.425	0.229	0.25	0.062	1.015	0.053**
Marital status								
Single*	1				1			
Married	1.17	0.324	4.228	0.811	2.73	0.526	14.122	0.232
Separated	1.07	0.099	11.433	0.957	3.34	0.186	59.926	0.413
Occupation								
Trader*	1				1			
Handwork	0.31	0.080	1.196	0.089	0.14	0.028	0.738	0.020**
Farmer	0.17	0.052	0.529	0.002**	0.09	0.022	0.337	0.000**
Unemployed	0.51	0.113	2.297	0.380	0.32	0.043	2.324	0.257
Alcohol consumption								
Yes*	1				1			
No	1.44	0.535	3.876	0.470	2.78	0.702	10.968	0.145
Been diagnosed of STIs								
Yes*	1				1			
No	0.85	0.313	2.311	0.751	0.85	0.263	2.732	0.783
Family history of hepatitis B								
Yes*	1				1			
No	0.19	0.059	0.612	0.005**	0.09	0.021	0.416	0.002**
Have multiple sexual partners								
Yes*	1				1			
No	1.18	0.454	3.051	0.737	2.11	0.679	6.587	0.196

*Reference; CI, confidence interval; STIs, sexually transmitted infections. **Significance at ≤0.05.

For the adjusted model (Table 3), after adjusting for (age, sex, marital status, occupation, alcohol consumption, family history of hepatitis B, history of being treated for STIs, and multiple sexual partners), compared to participants who were traders, there were statistically significant lower risk of hepatitis B infections in those who had handwork (i.e., mason, seamstress, head dressers) (OR = 0.14; 95% CI: 0.028–0.738; *p* = 0.020) and in those who were farmers (OR = 0.09; 95% CI: 0.022–0.337; *p* = 0.000). Compared to participants with family history of hepatitis B, there were statistically significant lower risk of hepatitis B infections (OR = 0.09; 95% CI: 0.021–0.416; *p* = 0.002) in those without a family history of hepatitis B (Table 3).

## 4 Discussion

This study assessed the prevalence and associated risk factors of HBV infection among adults in rural communities in the UER of Ghana. Findings provide important insights into the burden of HBV infection and highlight key socio-demographic and behavioral factors associated with the infection in this underserved population. The HBV prevalence of 9.63% observed in this rural Ghanaian population is consistent with the high endemicity documented in Ghana (8.48%) (11), across West Africa (>10%) (26), and substantially exceeds estimates reported from rural settings in India (2.5%) (27) and Brazil (~4.7%) (28). These differences highlight persistent geographic and structural inequities in HBV transmission, particularly in underserved rural communities. Collectively, the evidence suggests that while HBV remains a global health issue, its burden is disproportionately concentrated in rural and resource-limited settings such as Ghana (26). This underscores an urgent need for targeted HBV screening, vaccination, and community-based interventions to control and prevent ongoing transmissions in rural communities.

### 4.1 Socio-demographic and behavioral factors and HBV infection

Occupation emerged as a significant determinant of HBV infection. Participants engaged in farming and handwork were found to have significantly lower odds of HBV infection compared to traders, even after adjusting for confounding factors. While being consistent with previous findings (3, 17), this finding may reflect differences in social mobility, interaction patterns, and potential exposure networks among occupation groups. For example, traders may have increased contact with diverse populations through travel and commerce, which may potentially elevate their risk of HBV exposure. Conversely, farmers, who often work within localized settings, may have relatively lower exposure to high-risk interactions.

Sex was marginally associated with HBV infection, with females showing a lower likelihood of infection compared to males in the adjusted model. This finding is consistent with current evidence suggesting that males may be at higher risk of HBV infection due to genetic make-up (29, 30) and behavioral factors such as higher engagement in high-risk sexual practices, greater alcohol consumption, and lower healthcare utilization (20, 31, 32). The finding suggests that sex-specific behavioral and socio-cultural factors may influence HBV transmission dynamics in rural Ghana.

A family history of hepatitis B was one of the strongest predictors of HBV infection identified in this study. Consistent with previous findings (20, 33), participants without a family history of HBV had significantly lower odds of infection compared to those reporting a positive family history. This finding underscores the importance of intrafamilial and household transmission in rural settings, where close living arrangements, shared personal items, and limited awareness of HBV transmission pathways are common (33). Household transmission through contact with contaminated items such as toothbrushes, towels, and bedding remains a critical but often overlooked route of HBV spread in endemic rural communities (21, 22, 33).

Although behavioral factors such as multiple sexual partners, history of STIs, and alcohol consumption were included in the multivariable model, they did not demonstrate independent statistical significance in the adjusted model. Nevertheless, the relatively high proportion of participants reporting multiple sexual partners (36.7%) is not surprising because marriage is predominantly polygamous in the study area (34). However, this finding remains a public health concern because behaviors such as having multiple sexual partners are recognized risk factors for HBV transmission and may contribute to sustained community-level transmission in endemic settings (35). The lack of statistical significance may be attributable to the modest sample size or underreporting due to social desirability bias (36).

### 4.2 Implications for public health practice

The findings of this study highlight the urgent need for targeted, community-based HBV interventions in rural areas of the UER and beyond. Strategies should prioritize household-level screening, contact tracing, and vaccination of family members of infected individuals. Occupationally mobile groups, such as traders, should be specifically targeted for education and screening initiatives. Initiatives such as market-based outreach, peer-led education, and integrated screening models are critical strategies to improve uptake of HBV testing and vaccination among occupational mobile groups. Integrating HBV testing and vaccination into the newly launched primary healthcare initiative in Ghana and community outreach programs may significantly reduce the burden of HBV infection and interrupt transmission in rural communities.

### 4.3 Strengths and limitations of the study

#### 4.3.1 Strengths

This study has several notable strengths. It provides community-level evidence on the prevalence and risk factors of HBV infection in rural communities, a population that is often underrepresented in HBV research. By focusing on rural residents rather than urban or facility-based samples, the findings better reflect the true burden and transmission dynamics of HBV in underserved settings. In addition, the use of HBsAg testing to determine HBV status enhanced the accuracy of prevalence estimates and minimized misclassification bias associated with self-reported infection status. The application of multivariable logistic regression also allowed for identification of independent predictors of infection while controlling for potential confounders, which strengthened the validity of the findings.

#### 4.3.2 Limitations

Despite these strengths, the study has important limitations. The cross-sectional study design limits the ability to establish causal relationships between identified risk factors and HBV infection. The use of secondary data constrained analysis to variables captured during the community screening and did not allow assessment of important factors such as HBV vaccination status, duration of infection, viral load, or participants’ knowledge of HBV. The relatively small sample size may have reduced statistical power to detect significant associations for some behavioral factors. In addition, behavioral variables such as sexual practices and alcohol consumption were self-reported and may be subject to recall or social desirability bias. Finally, the findings may have limited generalizability beyond the study communities, as the data were drawn from selected rural areas within a single district.

## 5 Conclusion

Overall, this study provides valuable evidence that HBV infection remains a major public health challenge in rural UER of Ghana. The identification of occupation and family history as key risk factors emphasizes the need for context-specific prevention strategies that address both social and household transmission dynamics. Strengthening rural HBV control efforts is critical to achieving national and global hepatitis elimination goals.

## Data Availability

The data analyzed in this study is subject to the following licenses/restrictions: Hospital base dataset. Requests to access these datasets should be directed to princeapuri87@gmail.com.
