# Supplementary material for: Prevalence and risk factors of hepatitis B infection in rural communities: a cross-sectional analysis
Source: Front Public Health. 2026 Oct 13;14:1889741. doi: 10.3389/fpubh.2026.1889741 (PMC13710272; doi:10.3389/fpubh.2026.1889741)
Supplement: Supplementary file 1 [file Data_Sheet_1.PDF]

**Bembisi Community Health Screening Questionnaire (BCHSQ)**

**Participant #:** ..........................................................................................................

1. Age: ..........................................................................................................

2. Weight (Kg): ..........................................................................................................

3. Height (cm): ..........................................................................................................

4. BP: ..........................................................................................................

5. Blood glucose: FBS .............................................. RBS ..............................................

6. HBsAg: +Ve ☐ -Ve ☐

**Tick the box that applies to you**

7. Gender: Male ☐ Female ☐

8. Marital Status: Single ☐ Married ☐ Separate/Widow ☐

9. Educational level: No formal education ☐ Primary ☐ JSS/JHS ☐

SSS/SHS/Technical vocation ☐ Tertiary ☐

10. Community: Kandiga ☐ Mirigu ☐ Nabango ☐ Sirigu ☐

11. How would you evaluate your current financial status?

Much less than adequate ☐ Less than adequate ☐ Just enough ☐

More than adequate ☐ Much more than adequate ☐

12. Number of children: 1 – 2 ☐ 3 - 4 ☐ 5 - 6 ☐ 7 or more ☐ None ☐

13. How frequently do you see a healthcare provider? Every month ☐ Every 3 months ☐ Every 6 months ☐ Yearly ☐ Only when sick ☐

14. Do you smoke? Yes ☐ No ☐

15. If yes, how often do you smoke?

Once daily ☐ Three times daily ☐ Five times daily ☐ More than five times daily ☐

16. Do you drink alcohol or "Apatasi" or "Happy Bitters? Yes ☐ No ☐

17. If yes, how much alcohol do you drink?

1-3 glasses/Sackets daily ☐ 1-3 glasses/Sackets every 3 days ☐ 8 glasses/Sackets a week ☐ More thank 8 glasses/Sackets a week ☐

18. How often do you eat KooKo or drink Tea or Millo with sugar? Every morning ☐ Once every 3 days ☐ Once in 2 weeks ☐ 1-3 times in 3 months ☐ Never ☐

19. Do you complain of lack of salt or ask for more salt into your food? Yes, most times ☐ Yes, sometimes ☐ No ☐

20. Do you cook or does your family cook food with “Maggie”?

Yes, most times ☐ Yes, sometimes ☐ No ☐

21. “Dawadawa” or “Amaani” are ALWAYS part of our family meals. True ☐ False ☐

22. Do you eat tobacco or cola nuts? Yes ☐ No ☐

23. If yes, how often do you eat tobacco or cola nuts?

Once daily ☐ Three times daily ☐ Five times daily ☐ More than five times daily ☐

24. Do you share bath towel with others in the house? Yes ☐ No ☐

25. Do you share toothbrush with others in the house? Yes ☐ No ☐

26. Have you ever been diagnosed of or treated for STIs? Yes ☐ No ☐

27. Do you have a family history of hypertension or stroke? Yes ☐ No ☐

28. Do you have a family history of diabetes? Yes ☐ No ☐

29. Do you have a family history of liver disease or hepatitis B or Jaundice? Yes ☐ No ☐

30. Do you have a family history of anemia? Yes ☐ No ☐

31. Do you have a past history of smoking or alcohol use? Yes ☐ No ☐

32. Have you ever been transfused blood? Yes ☐ No ☐

33. Have you ever had a sexual partner who have other sexual partners? Yes ☐ No ☐

34. Describe your activity level in a typical day. Active ☐ Not active ☐

35. What work do you do for a living? Trading ☐ Hand work ☐ Gov. Worker ☐ Farmer ☐ No Work ☐ Student ☐

36. “Dawadawa” or “Amaani” are NOT always part of our family meals. True ☐ False ☐

37. In a day, I do sit more than I walk. True ☐ False ☐

38. How often do you take treatment for Malaria? Every 1-3 months ☐ Every 6 months ☐

Every year ☐ Never treated Malaria in the last 1-2 years ☐
